# EUPATI Guidance for Patient Involvement in Medicines Research and Development (R&D); Guidance for Pharmaceutical Industry-Led Medicines R&D

**DOI:** 10.3389/fmed.2018.00270

**Published:** 2018-10-09

**Authors:** Kay Warner, Wolf See, David Haerry, Ingrid Klingmann, Amy Hunter, Matthew May

**Affiliations:** ^1^GlaxoSmithKline, Brentford, United Kingdom; ^2^BAYER, Berlin, Germany; ^3^European Aids Treatment Group, Brussels, Belgium; ^4^European Forum for Good Clinical Practice, Brussels, Belgium; ^5^Genetic Alliance UK, London, United Kingdom; ^6^European Patients Forum, Luxembourg, Luxembourg

**Keywords:** patient engagement, guidance, patient involvement, pharmaceutical industry-led medicines R&D, medicines development, EUPATI

## Abstract

The importance and merits of greater patient involvement in medicines research and development (R&D) are commonly acknowledged and are thought to offer benefits for all involved parties. It helps to improve discovery, development, and evaluation of new effective medicines, based on the collaborative identification and understanding of unmet needs, research priorities, optimization of clinical study design, outcome measures, and endpoint development. It can result in increased transparency, trust and mutual respect between patients and other stakeholders. This applies to all stages of medicines R&D, from industry-led research, to regulation and licensing of medicines, to appraisal by health technology assessment (HTA) bodies. Integration of patients into the medicines development process needs to be structured and governed by clear rules and modes of operation to be effective and yield the best results for all stakeholders. Existing codes of practice for patient involvement with various stakeholders do not comprehensively cover the full scope of R&D, with the exception of more general statements applicable to interaction. Overarching guidance on meaningful and ethical interaction is missing. One specific aim of the European Patients' Academy on Therapeutic Innovation (EUPATI) was to close this gap through the development of guidance documents for selected stakeholders. Four separate guidance documents were developed, incorporating the results from comprehensive internal and external consultation. They cover patient involvement in: pharmaceutical industry-led medicines R&D; ethics committees; regulatory authorities; HTA. Each guidance suggests where patient involvement could be adopted or strengthened. The EUPATI guidance document for patient involvement in industry-led medicines research and development covers the interaction between patients and the pharmaceutical industry within all functions throughout the medicines R&D lifecycle in relation to medicines for human use. It relates to activities pre-approval and post approval, involving individuals and groups of patients. The guideline distinguishes between the level of expertise in a disease area that is required and the different areas where patient involvement can take place; however, this is not meant to limit involvement, and these opportunities may change and increase over time. This EUPATI guidance document is aimed at the pharmaceutical industry who want to engage patients in R&D activities, however all stakeholders involving patients in pharmaceutical-led medicines R&D should understand and use this EUPATI guidance document.

## Introduction

EUPATI started as a project of the Innovative Medicines Initiative (IMI) Joint Undertaking and from 1 February 2017 continues as a pan-European public-private partnership programme of the European Patients' Forum (EPF). Patient organizations, universities, not-for-profit organizations, and pharmaceutical companies are represented in the partnership. EUPATI focuses on education and training to increase the capacity and capability of patients to understand and contribute to medicines R&D, enabling broader, meaningful patient involvement in all its steps as well as providing objective, reliable, patient-friendly information for the public. EUPATI does not cover disease-specific issues or therapies, but the general process of medicines development. To find out more visit www.eupati.eu/.

As a result of the growing interest and willingness of patients to actively contribute to medicines development, more and more patients are interested in becoming involved in pharmaceutical industry-led medicines R&D across all functions throughout the medicines R&D lifecycle from pre-approval to post marketing activities. Similarly, there is a call within the pharmaceutical industry for earlier and greater involvement of patients in the medicines development although instances of patient engagement predominantly occur in clinical development and the creation of information for patients.

It is evident that patient involvement requires clear rules, determining procedures and ways of working to be effective, especially where patients and their organizations collaborate with industry as the interaction and potential for conflict of interest is under scrutiny. Existing codes of practice for patient involvement with various stakeholders do not comprehensively cover the full scope of R&D. Overarching guidance on meaningful and ethical interaction is missing. One specific aim of EUPATIwas to close this gap through the development of guidance documents for key stakeholders in order to support the development of efficient patient involvement across the whole cycle of medicines R&D.

Four separate guidance documents were developed by the multi-stakeholder EUPATI public-private consortium, based on the discussions held within the EUPATI project team, through its two patient involvement workshops with various stakeholders and incorporating the results from comprehensive internal and external consultation. They cover patient involvement in: Pharmaceutical industry-led medicines R&D; Ethics committees; Regulatory processes; and HTA.

The EUPATI guidance document in this article aims at providing recommendations for ground rules and proposals for the integration of patient involvement across the entire process of medicines R&D in the pharmaceutical industry and outlines specific activities where patients can be involved and influence future medicines research and development.

## The EUPATI guidance for patient involvement in industry-led medicines r&d

### Overarching principles for patient involvement throughout the medicines research and development process

EUPATI is a pan-European Innovative Medicines Initiative (IMI) project of 33 organizations with partners from patient organizations, universities, not-for-profit organizations, and pharmaceutical companies. Throughout EUPATI the term “patient” references all age groups across conditions. EUPATI does not focus on disease-specific issues or therapies, but on process of medicines development in general. Indication-specific information, age-specific or specific medicine interventions are beyond the scope of EUPATI and are the remit of health professionals as well as patient organizations. To find out more visit www.eupati.eu/.

The great majority of experts involved in the development and evaluation of medicines are scientists working both in the private and public sector. There is an increasing need to draw on patient knowledge and experience in order to understand what it is like to live with a specific condition, how care is administered and the day-to-day use of medicines. This input helps to improve discovery, development, and evaluation of new effective medicines.

Structured interaction between patients of all age groups and across conditions, their representatives and other stakeholders is necessary and allows the exchange of information and constructive dialogue at national and European level where the views from users of medicines can and should be considered. It is important to take into account that healthcare systems as well as practices and legislation might differ.

We recommend close cooperation and partnership between the various stakeholders including healthcare professionals' organizations, contract research organizations, patients' and consumers' organizations^*^^1^, academia, scientific and academic societies, regulatory authorities and health technology assessment (HTA) bodies and the pharmaceutical industry. Experience to date demonstrates that the involvement of patients has resulted in increased transparency, trust and mutual respect between them and other stakeholders.

It is acknowledged that the patients' contribution to the discovery, development and evaluation of medicines enriches the quality of the evidence and opinion available (1).

Existing codes of practice for patient involvement with various stakeholders do not comprehensively cover the full scope of R&D. The EUPATI guidance documents aim to support the integration of patient involvement across the entire process of medicines research and development. These guidance documents are not intended to be prescriptive and will not give detailed step-by-step advice.

EUPATI has developed these guidance documents for all stakeholders aiming to interact with patients on medicines R&D. Users may deviate from this guidance according to specific circumstances, national legislation or the unique needs of each interaction. This guidance should be adapted for individual requirements using best professional judgment.

There are four separate guidance documents covering patient involvement in:

- Pharmaceutical industry-led medicines R&D
- Ethics committees
- Regulatory authorities
- HTA.

Each guidance suggests areas where at present there are opportunities for patient involvement. These guidance documents should be periodically reviewed and revised to reflect evolution.

### This guidance covers patient involvement in industry-led medicines R&D

The following values are recognized in the guidance documents and worked toward through the adoption of the suggested working practices. The values are:

**Table:** 

Relevance	Patients have knowledge, perspectives and experiences tdat are unique and contribute to essential evidence for industry-led R&D.
Fairness	Patients have the same rights to contribute to the medicines R&D process as other stakeholders and have access to knowledge and experience that enable effective engagement.
Equity	Patient involvement in medicines R&D contributes to equity by seeking to understand the diverse needs of patients with particular health issues, balanced against the requirements of industry.
Capacity building	Patient involvement processes address barriers to involving patients in medicines R&D and build capacity for patients and research organizations to work together.

All subsequently developed guidances should be aligned with existing national legislation covering interactions as stated in the four EUPATI guidance documents.

### Disclaimer

EUPATI has developed these guidances for all stakeholders aiming to interact with patients on medicines R&D throughout the medicines R&D lifecycle.

These guidance documents are not intended to be prescriptive and will not give detailed step-by-step advice.

These guidances should be used according to specific circumstances, national legislation or the unique needs of each interaction. These guidances should be adapted for individual requirements using best professional judgment.

Where this guidances offers advice on legal issues, it is not offered as a definitive legal interpretation and is not a substitute for formal legal advice. If formal advice is required, involved stakeholders should consult their respective legal department if available, or seek legal advice from competent sources.

EUPATI will in no event be responsible for any outcomes of any nature resulting from the use of these guidances.

The EUPATI project received support from the Innovative Medicines Initiative Joint Undertaking under grant agreement n° 115334, resources of which are composed of financial contribution from the European Union's Seventh Framework Programme (FP7/2007-2013) and EFPIA companies.

## Specific guidance for patient involvement in industry-led medicines R&D

The importance and merits of greater patient involvement in medicines R&D is commonly acknowledged. A joint call for action to partner with patients in the development and lifecycle of medicines has been made by many pharmaceutical leaders (2). The patient community likewise has called for many years for companies to embed patient involvement in medicines R&D from the earliest stages (3).

There is an industry-wide movement toward patient focus, with the creation of the Patient-Centered Outcomes Research Institute (PCORI), FDA's Patient-Focused Drug Development (PFDD) initiative, Clinical Trials Transformation Initiative (CTTI) and the Patient Focused Medicine Development (PFMD) coalition. In Europe EUPATI and other IMI projects are leading the effort of generalizing patient involvement in R&D beyond specific indications. Greater patient engagement may offer many benefits for all involved parties, including the identification and understanding of unmet needs, research priorities, optimization of clinical study design and outcome measures and endpoint development. The goal of any interaction should be to improve medicines R&D by incorporating patient needs and priorities.

The need for clear guidance on patient involvement in industry driven R&D and interaction between patients and industry is based on the following:

- Existing codes of conduct reviewed do not thoroughly describe the involvement of patients in industry-led R&D, with exception of more general statements applicable to interaction.
- Overarching guidance on meaningful and ethical interaction is missing
- Patients and patient organizations should be involved proactively and longitudinally, especially during early discovery, development and post-approval stages of a medicine and interaction should not be confined to clinical development
- Language needs to be more directive toward patient involvement with a clear default statement that interaction is allowed unless expressly forbidden together with detailed agreement on how activities should be conducted.
- All interactions with patients should be conducted professionally, ethically and in a non-promotional manner (subject to local regulations).

### Codes of practice reviewed

A number of recognized codes were reviewed and provided an important foundation for this guidance document.

- The ECAB Protocol [description of and working procedures of ECAB (European Community Advisory Board, scientific working group at EATG, established 1997)]
- Mandate, objectives and rules of procedure for the European Medicines Agency Human Scientific Committees' Working Party with Patients' and Consumers' Organisations (PCWP) (30 May 2013)
- Minutes of EMA Human Scientific Committees' Working Party with Patients' and Consumers' Organisations (PCWP) meeting with all eligible organizations (31 January 2014)
- 10 December 2009 EMA Reflection Paper on the Further Involvement of Patients and Consumers in the Agency's Activities
- EMA leaflet on working with patients and consumers (updated 22/4/2015)
- EMA framework of interaction (revised 16 October 2014)
- Recommendations from ECAB meeting held in Bergen, Norway 1997 EATG ECAB, “The impatient Patient - From Anger to Activism” A systematic review of the history, working models, relevance and perspectives of the European Community Advisory Board
- FDA Patient Representative Program
- FDA Patient-Focused Drug Development; The Voice of the Patient: A Series of Reports from FDA's Patient-Focused Drug Development Initiative
- FDA Patient-Focused Drug Development: Enhancing Benefit-Risk Assessment in Regulatory Decision-Making
- WMA Declaration of Helsinki - Ethical Principles for Medical Research Involving Human Subjects.

### Scope

**Figure 1:**
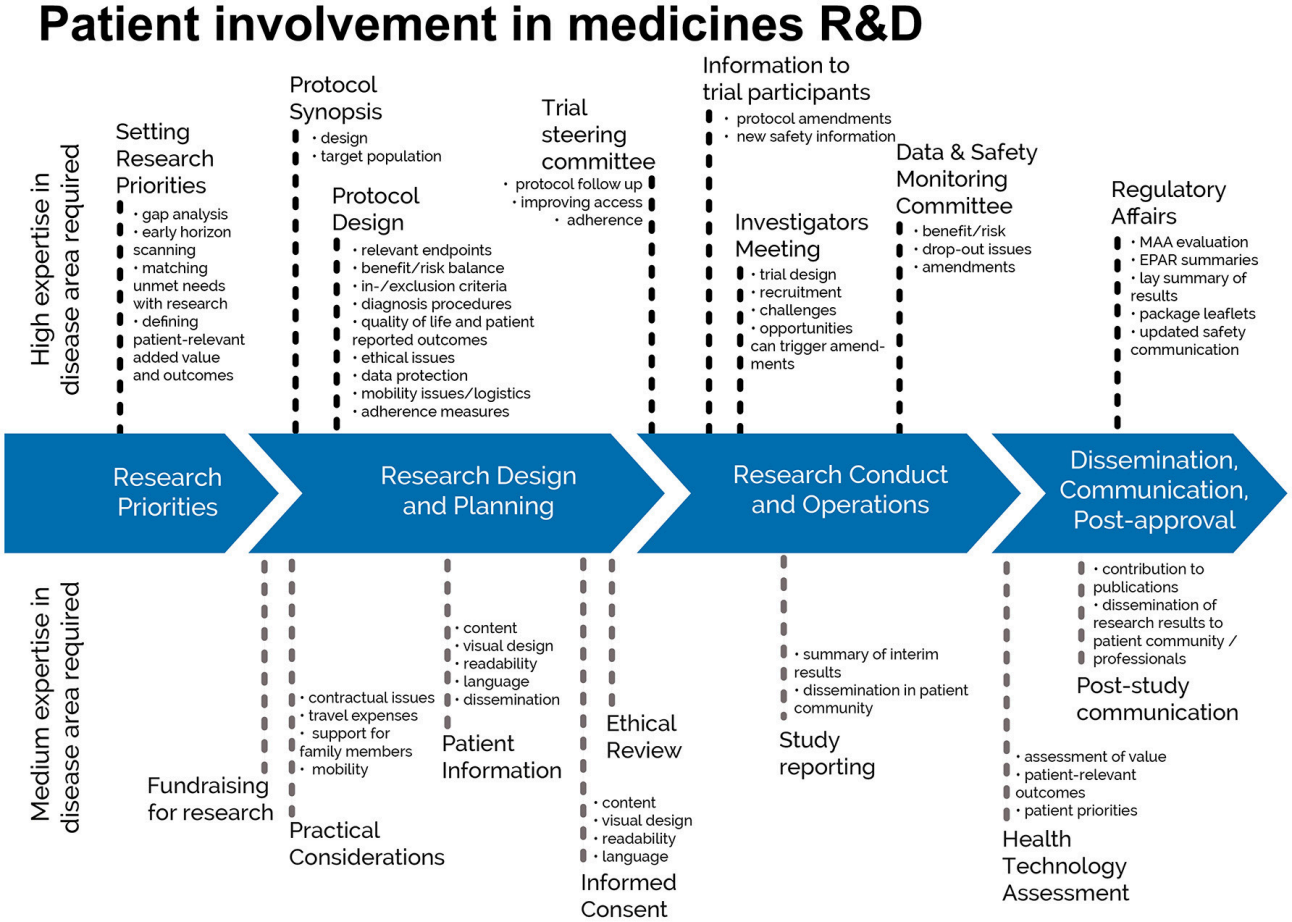
Patient involvement in medicines R&D. Patients can be involved across the process of medicines R&D. This diagram created by Geissler et al. (4) identifies some existing areas in which patients are involved in the process. It distinguishes between the level of expertise in a disease area that is required and the different areas where involvement can take place. There are individual cases where successful integration of patient input into medicines R&D have been demonstrated (5). Copyright: EUPATI, under a Creative Commons licence. Used with permission.

This European guidance covers the interaction between patients and the pharmaceutical industry throughout the medicines R&D lifecycle in relation to medicines for human use. This European guidance is for all functions in industry R&D on patient involvement throughout the medicines R&D lifecycle. This relates to activities pre-approval and post approval, involving individuals and groups of patients. “Patients” can be individual patients or their careers, or representatives from patient organizations with relevant expertise. See Figure 1 which indicates where patients can be involved currently; however this is not meant to limit involvement, and these opportunities may change and increase over time.

All activities should be in line with existing EU and national legislation covering pharmaceutical industry and interaction with the public. In addition, companies should follow their own internal procedures.

### Defining “patient”

The term “patient” is often used as a general, imprecise term that does not reflect the different types of input and experience required from patients, patient advocates and patient organizations in different collaborative processes.

In order to clarify terminology for potential roles of patient interaction presented in this and the other EUPATI guidance documents, we use the term “patient” which covers the following definitions:

- “Individual Patients” are persons with personal experience of living with a disease. They may or may not have technical knowledge in R&D or regulatory processes, but their main role is to contribute with their subjective disease and treatment experience.
- “Carers” are persons supporting individual patients such as family members as well as paid or volunteer helpers.
- “Patient Advocates” are persons who have the insight and experience in supporting a larger population of patients living with a specific disease. They may or may not be affiliated with an organization.
- “Patient Organization Representatives” are persons who are mandated to represent and express the collective views of a patient organization on a specific issue or disease area.
- “Patient Experts”, in addition to disease-specific expertise, have the technical knowledge in R&D and/or regulatory affairs through training or experience, for example EUPATI Fellows who have been trained by EUPATI on the full spectrum of medicines R&D.

There may be reservations about involving individual patients in collaborative activities with stakeholders on grounds that their input will be subjective and open to criticism. However, EUPATI, in line with regulatory authorities, instills the value of equity by not excluding the involvement of individuals. It should be left to the discretion of the organization/s initiating the interaction to choose the most adequate patient representation in terms of which type of patient for which activity. Where an individual patient will be engaged, it is suggested that the relevant patient organization, where one exists, be informed and/or consulted to provide support and/or advice.

The type of input and mandate of the involved person should be agreed in any collaborative process prior to engagement.

### Transparency

To increase transparency of patient involvement in industry-led medicines R&D, companies and patient organizations should, where allowed, publicly disclose their collaborative activities on an annual basis through their websites. Individual patient names and other protected health information should not be disclosed.

In some areas, the number of experienced and knowledgeable people might be small. This fact should not prevent consultation and building on this knowledge through parallel interactions with other interested parties (such as regulatory authorities, other pharmaceutical companies) however these interactions should be disclosed.

### Suggested working practices

Fostering and establishing long-term partnerships between patients, patient organizations and industry is the best approach to deliver benefits for all parties and is to be encouraged whilst respecting the independence of patients/patient organizations and other provisions set out in existing codes of conduct which would find their representation in robust, transparent operating procedures. However, it is recognized that relationship building may start with *ad hoc* interactions to meet short-term needs, but ideally transition to more frequent interactions as partnerships are established.

Internal cross-functional coordination in each pharmaceutical company for patient involvement would be very beneficial to all concerned, with a defined liaison role.

Pre-engagement discussions should take place to ensure mutually beneficial interaction and adequate preparation. Specific details regarding the interaction including scope, type of interaction, resource requirements and timelines should be agreed upon between patients, patient representatives and industry before interaction begins and defined in a written agreement.

### Defining the interaction

Patients, patient representatives and industry should take responsibility to ensure interactions are meaningful by clearly defined processes and actions, progressed to timelines. In addition, all participants should be prepared for the interaction.

Prior to each interaction, agree mutually on (where applicable):

- the objective of the project involving patients and/or areas of common interest to establish an agreed structured interaction, providing all parties with necessary protection with regards to independence, privacy, confidentiality and expectations
- the type of input and mandate of the involved person
- the tools and methods of interaction, e.g., types and frequency of meetings, ground rules, conflict resolution, evaluation
- desired patient/patient partner organization to foster long-term working partnerships, with independence ensured (in scope)
- the profile of the type of patient/s or patient representative/s to be involved and their number
- how activity outputs will be used and ownership of outputs
- how and when the patient/s involved will be informed of outcomes
- contractual terms and conditions including consent and compensation
- other elements according to the specific project.

## Patient identification/interaction

There are many ways to identify patients to be involved in an interaction. The main routes are through:

- existing patient organizations
- EUPATI or similar project
- advertising opportunities for patient participation
- existing relationships with healthcare providers, hospitals and researchers and other agencies
- unsolicited requests previously made by interested parties
- existing advisory boards/groups (e.g., EFPIA Think Tank, Patients and Consumers Working Party at the EMA)
- third party agencies

## Compensation

It should be recognized that in many situations patients involved in activities do so voluntarily either as an individual but also when a member of an organization. Consideration should therefore be given to:

- compensate for their total time invested plus expenses.
  - any compensation offered should be fair and appropriate for the type of engagement. Ideally travel costs would be paid directly by the organizing partner, rather than being reimbursed.
- covering the costs incurred by patient organizations when identifying or supporting patients for involvement in activities (i.e., peer support groups, training and preparation) should also be considered.
- help organize the logistics of patient participation, including travel and/or accommodation.

Compensation also includes indirect benefits in kind (such as the patient organization providing services free of charge) or any other non-financial benefits in kind provided to the patient/patient organization (such as training sessions, agency services, the setting up of web sites).

All parties should be transparent about any compensation arrangements.

## Written agreement

At a minimum a written agreement should clearly define: a description of the activity and its objectives, the nature of the interaction during the activity, consent (if relevant), release, confidentiality, compensation, data privacy, compliance, declaration of conflict of interest, timelines. Interaction may only proceed on the basis of a written agreement that at a minimum spells out the basic elements of the collaboration (e.g., rules of engagement, compliance, intellectual property, financial payments).

Care should be taken so that written agreements are clear and do not limit appropriate knowledge sharing.

### Events and hospitality

The method of interaction (meetings, telephone discussions, etc.) should be discussed and mutually agreed, with convenience for patients/patient organizations as the main priority. If the interaction requires in person meetings or the development and delivery of events, these should follow existing codes of conduct, local legislation, in terms of appropriate venue/location and the level of hospitality provided.

When events are organized, the ability of any intended patient audience to attend should be considered, with appropriate measures taken to enable accessibility, assisted travel and entry into the event.

Appendices to the guidance are available in the online version of the guidance document (6).

## Discussion

The absence of an overarching guidance on meaningful and ethical interaction of patients in pharmaceutical industry-led medicines R&D was identified as a gap by the EUPATI project. There is no defined, recognized code of practice for patient involvement in R&D. This was confirmed following a review of existing codes of practice. There were general statements applicable to interaction, but the full scope of patient involvement in medicines R&D was not covered.

Based on the discussions held within the EUPATI project team as well as through its two patient involvement workshops with various stakeholders and input received during internal and external consultation, the multi-stakeholder EUPATI public-private consortium has developed criteria and conditions for the involvement of patients in pharmaceutical industry-led medicines R&D.

Advice on rates of compensation was requested during the consultation period for the EUPATI guidance but ultimately it was not possible to provide definitive detailed guidance. This is at least in part due to the widely varying rules and procedures of compensation for advisory or other contributions within projects, across countries, between companies or government institutions, or even systematic differences between what is considered a “fair” compensation scheme in a given societal context.

The consultation response was unable to answer the numerous questions around how to identify and involve patients in different capacities across the medicines R&D process. The diversity of possible roles patients could take impeded developing more detailed guidance. As stated in the guidance document, “it should be left to the discretion of the organization/s initiating the interaction to choose the most adequate patient representation in terms of which type of patient for which activity.” However, there are individual cases where successful integration of patient input into medicines R&D have been demonstrated.

Those who want to increase their patient engagement can use these examples as a basis for a considered approach, while following the suggested working practices provided in this EUPATI guidance document.

Involvement of patients in industry-led medicines R&D should occur across the entire medicines R&D lifecycle. EUPATI in its diagram (Figure 1) have indicated where the key involvement points are to answer one question that is frequently asked: when should patients be involved? The EUPATI definition of patient levels of expertise will further help to identify the most suitable patients for the different tasks in medicines R&D.

As stated in the Aurora Project's discussion paper (7), “Being patient centric is no harder than the difficult and expensive process of researching and proving the effectiveness of medicines.” Their survey results set out some benchmarks and provide, for the first time, an accurate picture of pharmaceutical industry patient-centric efforts and outcomes, highlighting specific case studies to support conclusions.

In the largest-ever survey of its kind, with insights gleaned from 2,346 respondents from 84 countries in a variety of pharma roles shows that pharma assesses being on the right track. They indicate that industry is making efforts to listen to patients, provide patient programmes, tools and education, place greater emphasis on leadership and organizational culture, and enhance clinical trial design. However, a lack of know-how was cited as one barrier, along with others including a belief in the traditional product-focused “push” model vs. investment in a novel patient centric model, the gradual but slow evolution of the regulatory environment, patient centricity not being prioritized, and insufficient budget or resources to enable systematic follow through on intentions.

In all four guidance documents, EUPATI set out guiding principles and suggested working practices with the intention that these will be adopted and further expanded and agreed to form a future code of practice which the industry can follow. Without a definitive and recognized code of practice, patient involvement may not advance at the rate at which both patients and the industry desire.

## Conclusion

The pharmaceutical industry should strive to involve patients early in medicines development, preferably before the clinical development phases. When a product under development has reached clinical development, many key decisions about a medicine have already been taken and cannot be reversed.

The infrastructure and governance for inclusion of patients in meaningful interaction requires further dedicated focus. Pharmaceutical companies need to continue to evolve their processes and governance infrastructure to integrate patients' involvement and patient organizations need to work on preparedness of their members to provide relevant input and increase their ability to identify individual patients who are interested in getting involved. Opportunities for involvement also need to be properly communicated by researchers to patients.

To break down the perceived barriers, pharmaceutical companies can build on the important foundation, key points and working practices recommended in this EUPATI guidance document or even call for an industry code of practice.

## Author contributions

KW drafted the article and the guidance document with significant input from WS and DH. WS, DH, AH, IK, and MM reviewed and revised the article and document.

### Conflict of interest statement

The authors declare that the research was conducted in the absence of any commercial or financial relationships that could be construed as a potential conflict of interest.

## Footnotes

^1^^*^Consumers are recognised as stakeholders in the healthcare dialogue. The scope of EUPATI focuses on patients rather than consumers. This is reflected in the educational material and guidance documents.
